# Supplementary material for: Three-dimensional strain dynamics govern the hysteresis in heterogeneous catalysis
Source: Nat Commun. 2020 Sep 18;11:4733. doi: 10.1038/s41467-020-18622-2 (PMC7501851; doi:10.1038/s41467-020-18622-2)
Supplement: Supplementary file 1 — Supplementary Information [file 41467_2020_18622_MOESM1_ESM.pdf]

Three-dimensional strain dynamics govern the hysteresis in heterogeneous catalysis

Aline R. Passos[1], Amélie Rochet[1*], Luiza M. Manente[1], Ana F. Suzana[1,2], Ross Harder[3], Wonsuk Cha[3] and Florian Meneau[1]*

[1] Brazilian Synchrotron Light Laboratory (LNLS), Brazilian Center for Research in Energy and Materials (CNPEM), 13083-970, Campinas, SP, Brazil. Correspondence and requests for materials should be addressed to aline.passos@lnls.br, amelie.rochet@lnls.br.

[2] Instituto de Química, UNESP, Rua Professor Francisco Degni, 14800-900 Araraquara, SP, Brazil.

[3] Advanced Photon Source, Argonne National Laboratory, 9700 South Cass Avenue, Argonne, IL 60439, USA.

Supplementary information

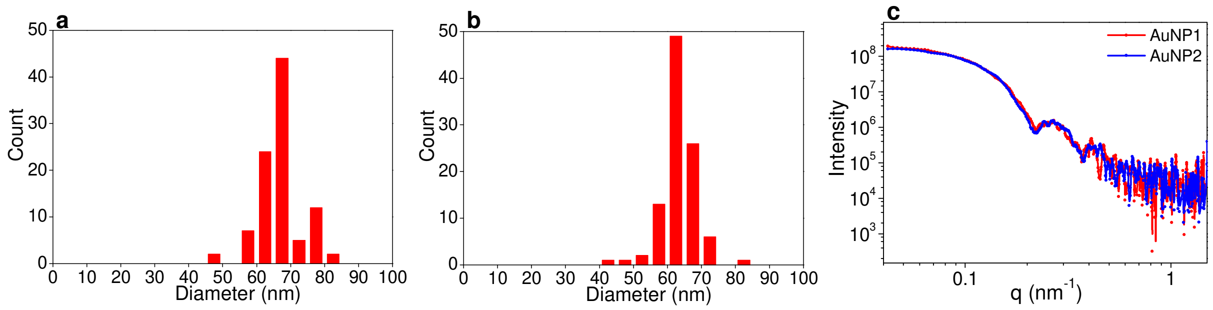

Supplementary Figure 1. Particle size distribution and dispersion. Particle size distribution obtained from STEM images of (a) cuboctahedric and (b) cubic shape nanoparticles. (c) Small Angle X-ray Scattering patterns of the gold suspensions, AuNP1 correspond to the cuboctahedra and AuNP2 to the cubes.

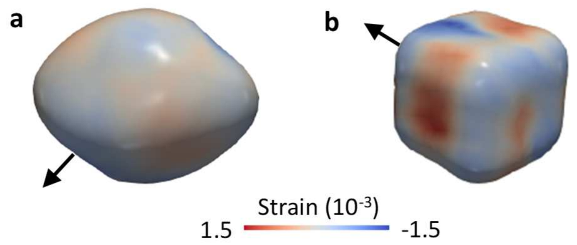

Supplementary Figure 2. **3D distribution of the strain at room temperature.** Strain field projected along (111) of the dried supported gold crystals from Figure 1: **(a)** cuboctahedron and **(b)** cube. Note that the sign of the [111] component of strain is largely determined by the angle between the crystal plane and the [111] crystalline direction.

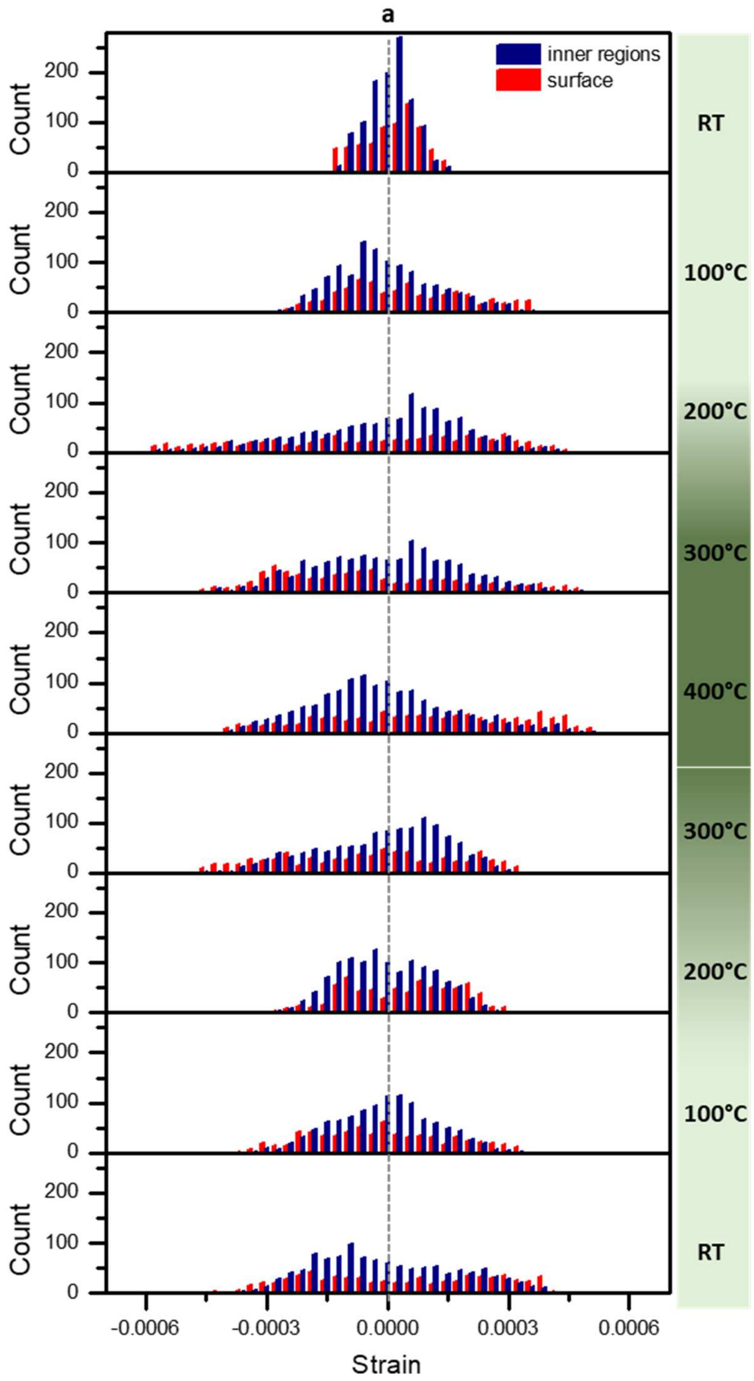
a
inner regions
surface
Count
200
100
0
RT
100°C
200°C
300°C
400°C
300°C
200°C
100°C
RT
-0.0006
-0.0003
0.0000
0.0003
0.0006
Strain

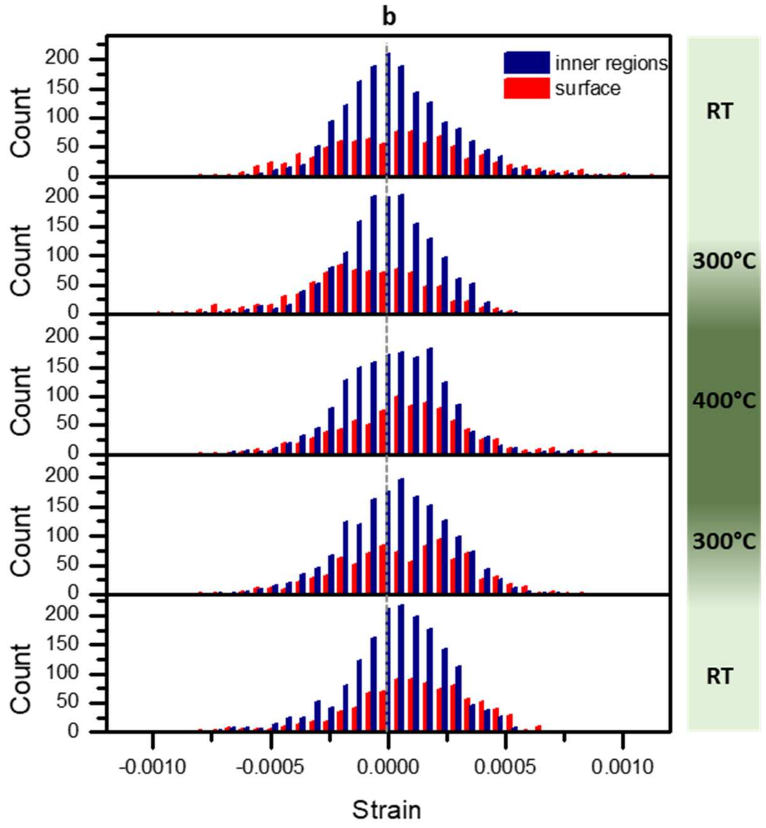

Supplementary Figure 3. **Statistical distribution of strain.** **(a)** AuNP1 and **(b)** AuNP2 during the hysteresis loop. The strain of the inner regions in blue of the nanocrystal is shown with the surfaces strain in red. The green gradient is illustrating the increase/decrease of catalytic activity with the temperature simultaneously followed by mass spectrometry to the BraggCDI experiment.
